# Pharmacokinetics, pharmacodynamics, efficacy and drug resistance selection of injectable long-acting lenacapavir pre-exposure prophylaxis (PrEP) against HIV

**DOI:** 10.1101/2025.08.26.25334527

**Published:** 2025-09-22

**Authors:** Hee-yeong Kim, Antonia Liebenberg, Lanxin Zhang, Max von Kleist

**Affiliations:** 1 Project group 5 ”Systems Medicine of Infectious Disease”, Robert Koch Institute, Berlin, Germany; 2 Mathematics for Data Science, Dep. of Mathematics and Computer Science, Freie Universität Berlin, Germany

## Abstract

Oral pre-exposure prophylaxis (PrEP) denotes an effective strategy to reduce the risk of HIV infection. However, many individuals encounter difficulties adhering to the once-daily regimen, which highlights the need for a broader portfolio of PrEP options. The novel HIV capsid inhibitor lenacapavir (LEN), when injected every six month, has shown potential in the recently completed clinical trials. However, clinical trials may not enable to accurately estimate prophylactic efficacy and protective concentration benchmarks. Moreover, since LEN may persist up to two years, there may be a risk for de novo resistance emergence after stopping PrEP.

We developed an integrated pharmacokinetic-pharmacodynamic (PK-PD) model of LEN and incorporated observed variability from Phase III clinical data into our analysis. The model was used to quantify prophylactic efficacy against wild type (WT) and resistant virus, as well as to quantify risks of de novo drug resistance emergence when LEN-PrEP is stopped.

We estimated a 95% preventive plasma concentration $\left(EC_{95}\right)$ of 5.8ng/mL. LEN fully prevented infection with WT virus at plasma concentrations above 10ng/mL, which in an ‘average individual’ is achieved within 155hours after the first subcutaneous injection of 927mg and persists for up to 45weeks after the last injection. However, considering PK variability indicates that >5.8 or >10ng/mL are not surpassed at all times in all individuals. Full protection against mutant strains carrying the Q67H and N74D mutations was achieved at plasma concentrations of 35ng/mL and 85ng/mL, whereas LEN concentrations of 3095ng/mL, 218ng/mL, 100ng/mL provided full protection against variants carrying Q67H+N74D, Q67H+T107N and Q67H+N74S. The mutant selection window for N74D and all double mutants overlapped with steady-state concentrations for twice-yearly dosing. De novo resistance emergence is possible once LEN concentrations fall below 10ng/mL after the last injection and this temporal window lasts for ≈ 201, 105, 79, 96 and 86days for the Q67H, N74D, Q67H+N74D, Q67H+T107N and Q67H+N74S mutants, respectively, in an ‘average individual’.

Our results highlight a substantial risk for de novo drug resistance emergence when LEN SC injection are stopped, calling for strategies to manage LEN discontinuation.

## Introduction

HIV remains a relevant public health treat, with globally 1.3 million new infections, 39.9 million people living with HIV (PWHIV) and 630,000 HIV-related deaths in 2023 [1]. While highly active antiretroviral treatment (HAART) can suppress virus replication and prevent acquired immunodeficiency syndrome (AIDS) [2], HIV persists in latently infected cells and may immediately rebound when treatment is stopped [3]. Unfortunately, there is no effective HIV vaccine and most vaccine trials had disappointing outcomes [4, 5]. While vaccine options are missing, many antivirals can also be used as pre-exposure prophylaxis (PrEP) to prevent HIV infection. HIV PrEP with once-daily oral tenofovir disoproxil fumerate and emtricitabine (TDF/FTC) is highly cost-effective [6], widely available and highly efficient in preventing HIV infection, when taken regularly [7]. However, adhering to the daily oral regimen poses challenges to some individuals, and in particular for heterosexual cis-women [8], which denote the most relevant group for HIV prevention globally [9]. For example, in recent PrEP clinical trials [10], as little as 16% of study participants adhered to oral TDF/FTC by week 52.

Long-acting (LA-)PrEP formulations might offer a solution to individuals struggling to adhere to a once-daily regimen. Bi-monthly injections with carbotegravir (CAB) [11, 12] proved efficient in preventing HIV infection in both MSM and cis-gender women and are available in many high-income countries. Trials with islatravir implants for PrEP have been put on halt, due to side effects [13], while, more recently, twice-yearly injections with long-acting lenacapavir (LEN) successfully completed Phase III clinical testing, indicating > 90% prophylactic efficacy [10, 14]. However, it is impossible to precisely quantify the extend of LEN prophylactic efficacy, because of statistical limitations of clinical PrEP studies, as well as limitations in their design [8, 15]. Moreover, the risk of drug resistance emergence cannot be systematically evaluated in clinical trials. While LA-PrEP offers the advantage of infrequent dosing, the long persistence of antiviral drugs may pose considerable risk to drug resistance selection, particularly when LA-PrEP is stopped due to side effects, lack of financial means, insurance coverage or unwillingness to continue PrEP. For example, CAB and LEN have plasma half-lives of ≈ 47 and 56–84 days, respectively [16, 17], implying that drug concentrations insufficient to protect from infection, but sufficient to select drug resistance [18], may persist 1–2 years after stopping LA-PrEP. The case of an individual who had to stop LA-CAB, because he fell out of social insurance, acquired HIV infection and subsequently developed de novo drug resistance should be interpreted as a warning in this context [19].

LEN is used as a salvage therapy in heavily treatment-experienced adults with multi-drug resistant HIV [20], because there are no known cross-resistances to other antiretrovirals [21, 22]. However, the use of LEN in salvage therapy makes evaluations regarding drug resistance selection and propagation particularly relevant.

LEN is a first-in-class inhibitor of HIV capsid function [23, 24] that is believed to interfere with capsid assembly and plasticity [25]. Oral and subcutaneous (SC) formulations of LEN are currently in clinical development, with two recommended initiation regimens available for treatment [26]. These involve an oral leading phase with 600mg LEN as tablets, followed by SC injections of 927mg every six months. In addition, a new intramuscular (IM) formulation is being investigated in a Phase I clinical trial for PrEP. This once-yearly regimen includes a 5000mg IM dose of LEN and is being evaluated in two formulations (containing 5% w/w and 10% w/w ethanol) [27]. LEN has a relatively low barrier to drug resistance and *in vitro* resistance selection experiments have identified seven major LEN-associated mutations (L56I, M66I, Q67H, K70N, N74D, N74S, T107N and their combinations) affecting LEN binding to the capsid protein [28–30]. Of those mutations, N74D was detected in 2/2 participants who got infected during the PURPOSE 2 study [14].

Currently, no mathematical model has been published to quantify prophylactic efficacy of LEN for different administration schemes, or for assessing the risk of drug resistance emergence in the context of LA-LEN PrEP. In order to fill this knowledge gap, we developed an integrated PK/PD model for LEN based on clinical data from oral administration and long-acting subcutaneous (SC) and intramuscular (IM) formulations. An HIV-1 viral dynamics model was used to estimate the antiviral potency $\left(IC_{50}\right)$ of LEN against wild type (WT) virus. We extended the viral dynamics model to account for the emergence and dynamics of drug-resistant variants utilizing *in vitro* phenotypic data [28]. We then evaluated the efficacy of long-acting LEN across a range of dosing regimen against wild type virus and transmitted drug resistance. Finally, we assessed the risk of de novo drug resistance emergence after wild type infection, in case when LA-LEN PrEP is stopped. Overall, our study provides a quantitative framework to assess the benefits and potential pitfalls of LEN with regards to PrEP and drug resistance selection.

## Methods

### Clinical PK data

We considered all relevant, publicly available pharmacokinetic data, as detailed in Table 1. In total, this dataset encompassed 15 different dosing schemes and three administration routes (oral, subcutaneous and intramuscular) from which we extracted mean and median pharmacokinetic data (Engauge Digitizer).

### LEN PK modeling

#### LEN Compartment Model.

Mean or median concentration–time data were fitted to a one-compartment pharmacokinetic (PK) model [31, 34] that can receive input from either oral administration, or one of the two parenteral routes following subcutaneous or intramuscular injection of LEN, Fig. 2A. In brief, LEN in blood plasma is eliminated with rate constant $k_{e}$. The oral route follows simple first-order absorption kinetics with rate $k_{a}$. Subcutaneous (SC, abdominal) or intramuscular (IM, gluteal) injection involves parallel release processes: after injection, a portion of the drug is released by a direct mechanism (with rate $k_{direct}$) from the soluble fraction of the formulation. The remaining part is released by an indirect mechanism (with rate $k_{indirect}$) through the formation of a solid depot at the injection site. As a result, part of the dose enters systemic circulation rapidly, while the solid depot dissolves gradually over time, ensuring steady drug levels over a prolonged period. To account for the delay in systemic availability (lag time), the indirect release pathway is modeled using n transit compartments subject to a gamma distribution [35]. All parameter estimates are listed in Table 2. The corresponding ODEs are given below.


(1) $$\frac{d}{dt}Oral=-k_{a}\cdot Oral\;(\text{oral dosing})$$

(2) $$\frac{d}{dt}PI=-\left(k_{direct}+k_{indirect}\right)\cdot PI\;\text{(parenteral dosing)}$$

(3) $$\frac{d}{dt}C=\left(k_{a}\cdot Oral+k_{direct}\cdot PI\cdot Frac\;(\text{plasma})\right.\;\left.\;+k_{indirect}\cdot PI\cdot (1-Frac)\cdot \frac{{\left(k_{indirect}\cdot t\right)}^{n}}{n!}\cdot e^{-k_{indirect}\cdot t}\right)/V_{d}-k_{e}\cdot C$$

#### LEN Oral PK.

The determination of the absorption rate constant, $k_{a}$, is based on the time to maximum concentration $\left(T_{max}\right)$ , assuming a one-compartment model with linear absorption and first-order elimination. Dose-dependent $T_{max}$ values were obtained from the respective PK study, see Supplement Table S1 [32]. The plot of the log-transformed PK data vs. time is linear, and the slope yields the elimination rate constant $k_{e}$. The absorption rate constant $k_{a}$ could then be numerically obtained from the following equation:

(4) $$T_{max}=\frac{ln\left(k_{a}\right)-ln\left(k_{e}\right)}{k_{a}-k_{e}}.$$

Plasma levels tended to increase with higher doses disproportionally with dose. Non-linear plasma levels were modeled with a dose-specific $V_{d}$ (dose) [34]. The absorption is reported to be unaffected by food (see [32]).

#### LEN Parenteral PK.

LEN exhibits flip-flop PK [34], characterized by a slower absorption compared to the elimination rate (i.e. $k_{direct}+k_{indirect}<k_{e}$). This makes absorption the rate-limiting factor of the terminal elimination phase. To maintain the kinetics, we fitted individual absorption parameters for the different parenteral formulations (three SC and two IM). This includes the fraction of drug directly entering systemic circulation (Frac) and the number of transit compartments $\left(n\right)$ to capture the lag time, but with consistent volume of distribution $V_{d}$ and elimination rate $k_{e}$. The SC-PK data show an approximately dose-proportional increase in exposure, whereas the available IM data were not sufficient to obtain a relationship. All parenteral formulations show distinct plasma pharmacokinetics (see Table 2). In each case, the indirect absorption pathway is much slower than the direct process, contributing to a prolonged half-life (shown in Supplementary Table S1).

#### Numerical simulation.

For numerical simulation of LEN pharmacokinetics, we solved the system of ordinary differential equations (eqs. (1)-(3)) with corresponding initial conditions $z_{0}=[\text{dose},0,0]$ for oral administration and $z_{0}=[0,\text{dose},0]$ for SC and IM administration using *scipy.integrate.solve_ivp()*, version 1.16.0.

Multiple doses were modeled by adding the dose at each pre-defined dosing event time $\tau_{j}$ to the dosing compartment D, depending on the route of administration; $z_{\tau_{j}^{+},D}=z_{\tau_{j}^{-},D}+\text{dose}$, where $\tau_{j}^{-}$ represents the time of the dosing event (before applying the dose) and $\tau_{j}^{+}$ after applying the dose.

#### Parameter estimation.

For oral dosing, we estimated a single dose-dependent parameter (volume of distribution $V_{d}$). Based on our analysis, this parameter can also be obtained by the power-law function f (dose) = 508.16 · dose^0.61^ with input dose in mg. For SC and IM dosing, 4 model parameters were estimated, including a common $V_{d}$ and fixed $k_{e}$. Parameters n, Frac and $k_{direct}$ for SC PEG/water formulation (Study 4) were initialized with values taken from an adapted two-compartment model [34]. All free parameters were optimized in a least-squares sense using *lmfit.minimize()*, version 1.3.3 until convergence to best-fit parameters occurred. Final parameter estimates are depicted in Table 2.

#### PK variability.

To account for inter-individual variability, we extracted data (Engauge Digitizer) from the Purpose 2 trial, which assessed 927 mg twice yearly SC injections and recorded concentration measurements across a random cohort of 10% of study participants at weeks 4, 8, 13, 26, 39 and 52 after the first Len injection [14]. Ratios of minimum and maximum concentrations relative to the median were calculated for these six time points, and the geometric mean of these ratios was then determined across all time points. This yielded a lower PK variability limit of 0.15 times the median concentration and a factor of 4.0 times the median concentration as an upper concentration limit. To depict PK variability, we applied these ranges (0.15× median, 4.0× median) to simulated data (SC and IM injections) from our PK model. An application of these ranges to data from Phase I single SC dose 927 mg injections is shown in Supplementary Figure S1 together with the original PK variability data from Purpose 2.

### LEN antiviral effects

**Fig 1.**
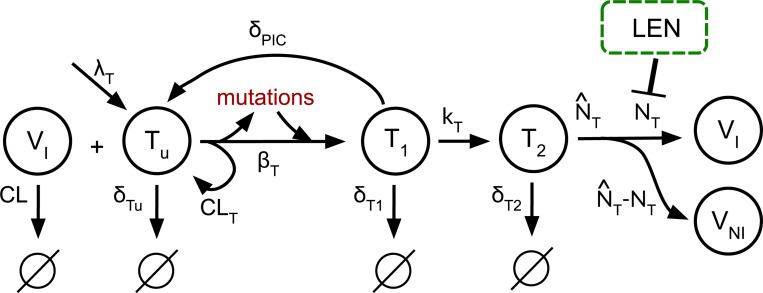
Simplified viral dynamics model incorporating the mechanisms of action of LEN and the emergence of mutated strains. A susceptible T-cell $\left(T_{u}\right)$ can be successfully infected by an infectious virus $\left(V_{I}\right)$, resulting in an early-infected T-cell $\left(T_{1}\right)$, which may transition into a late-infected T-cell $\left(T_{2}\right)$. Mutations (in red) can emerge during reverse transcription, which is captured by the transition of uninfected to early infected cells $V_{I}+T_{u}\to T_{1}$ in the model. LEN inhibits the production of infectious virus $\left(V_{I}\right)$ and leads to a proportional increase in the number of non-infectious virus $\left(V_{NI}\right)$.

Based on LEN’s main mechanism of action (MOA), we modeled LEN to interfere with the late phase of the HIV-1 replication cycle, similar to protease and maturation inhibitors [36]. I.e., in or model LEN would disrupt viral maturation, leading to the production of non-infectious viral particles, as shown in Fig. 1.

#### Estimation of antiviral potency.

We used viral load (VL) data from a Phase Ib proof-of-concept study, that assessed the mean log_10_-transformed change in plasma HIV-1 RNA/mL following SC aqueous suspension administration (Study 3) of 20–450 mg LEN in individuals with untreated HIV-1 infection [24]. The decline in VL following LEN monotherapy was modeled by linking the PK model to an established HIV-1 viral dynamics [36]. To model direct drug effects $\eta \left(C_{t},FC\right)$, we utilized a standard Emax model [37]:

(5) $$\eta \left(C_{t},FC(i)\right)=\frac{C_{t}^{m}}{C_{t}^{m}+{\left(FC(i)\cdot IC_{50}\right)}^{m}},$$

where $C_{t}$ denotes the plasma LEN at time t, $IC_{50}$ denotes the fifty percent inhibitory concentration against wild type and the Hill coefficient was set to m=2.1 [38]. FC(i)>1 denotes the fold-change (FC) for resistant strains i. We used values determined *in vitro* using a single-cycle assay in MT-2 cells [28], which were FC(L56I)=239, FC(M66I)>3200, FC(K70N)=24, FC(Q67H)=6.3 and FC(N74D)=22 as well as three double mutations FC(Q67H+T107N)=62, FC(Q67H+N74S)=32 and FC(Q67H+N74D)=1099 for the considered mutants. The viral dynamics parameters with $\beta_{T}=1.9\times 10^{-12}\;1/\text{day}$ and $\beta_{M}=1\times 10^{-14}\;1/\text{day}$ were adjusted to match the patients baseline VL of approximately 4.5 log_10_ HIV-1 RNA copies/mL in plasma. These parameters also align with the onset of the second-phase viral decay, both in the absence of LEN. The complete list of parameters can be found in [39]. After PK-PD coupling, the $IC_{50}$ was estimated in a least-squares sense.

### Viral replication and emergence of mutant strains

We simplified the viral dynamics model focusing on T-cells dynamics, as this is sufficient to estimate prophylactic efficacy [40]. Moreover, we extended this model to include the occurrence of mutant strains, as shown in Fig. 1.

In brief, when the virus enters the body, a susceptible target cell $T_{u}$ can be successfully infected by an infectious virus $V_{I}$, resulting in the formation of an early infected T-cell $T_{1}$. In the case where the virus is cleared without establishing infection the target cell remains uninfected and the virus is removed at a clearance rate $CL_{T}$. Following successful integration of the viral genome at rate $k_{T}$, early infected cells transition into late infected cells $\left(T_{1}\to T_{2}\right)$. LEN further interferes with the assembly of the capsid by inhibiting the rate $N_{T}$ at which new infectious virus particles are produced. This leads to increased formation of malformed, non-infectious virus particles $\left(V_{NI}\right)$. The rate of release of non-infectious virus is given by $\left({\hat{N}}_{T}-N_{T}\right)$. All viral dynamics parameters were taken from [39].

Mutation occur at the step of reverse transcription, which in our model is captured by transition of an uninfected cell $T_{u}$ to an early infected cell $T_{1}$ [36].

Uninfected T-cells are produced at birth rate $\lambda_{T}$, and the parameters $\delta_{T_{u}}$, $\delta_{T_{1}}$, $\delta_{PIC}$, and $\delta_{T_{2}}$ represent the death rate constants of the respective cell populations. A free infectious virus gets cleared at rate CL. An overview of the dynamics system is given by the following ODE-system:

(6) $$\frac{d}{dt}T_{u}=\lambda_{T}+T_{1}(i)\cdot \delta_{PIC}-T_{u}\cdot \delta_{T_{u}}-\sum_{i}V_{I}(i)\cdot \beta_{T}\cdot T_{u}$$

(7) $$\frac{d}{dt}T_{1}(i)=\sum_{k}V_{I}(k)\cdot p_{k\to i}\cdot \beta_{T}\cdot T_{u}-T_{1}(i)\cdot \left(\delta_{T_{1}}+\delta_{PIC}+k_{T}\right)$$

(8) $$\frac{d}{dt}T_{2}(i)=k_{T}\cdot T_{1}(i)-T_{2}(i)\cdot \delta_{T_{2}}$$

(9) $$\frac{d}{dt}V_{I}(i)=s(i)\cdot \left(1-\eta \left(C_{t},i\right)\right)\cdot N_{T}\cdot T_{2}(i)-V_{I}(i)\cdot \left(CL+\left(CL_{T}+\beta_{T}\right)\cdot T_{u}\right)$$

(10) $$\frac{d}{dt}V_{NI}=\sum_{i}\left(\left({\hat{N}}_{T}-s(i)\cdot \left(1-\eta \left(C_{t},i\right)\right)\cdot N_{T}\right)\cdot T_{2}(i)\right)-CL\cdot V_{NI}$$

The selective disadvantage s(i)<1 (1 - relative fitness) of a mutant strain i reflects the loss in replication ability of a resistant phenotype relative to the wild type virus [41]. We used values determined *in vitro* using a single-cycle assay in MT-2 cells [28], which were s(L56I)=0.91, s(M66I)=0.94, s(K70N)=0.93, s(Q67H)=0.05 and s(N74D)=0.52 and for three double mutations s(Q67H+T107N)=0.59 , s(Q67H+N74S)=0.66, and s(Q67H+N74D)=0.71. The probability that any strain i mutates into another strain k was given by the transition probability $p_{k\to i}$ and accounts for all possible states along the mutagenic pathway. We computed the transition probability based on the Hamming distance h(i,k) between strains i and k in terms of the number of amino acid substitutions required to convert one genotype into the other. Assuming that each position mutates independently, the transition probability is calculated as:

(11) $$p_{k\to i}=\mu^{h(i,k)}\cdot {\left(1-\mu \right)}^{N-h(i,k)}$$

where μ represents the mutation probability per base ($\mu \approx 2.16\cdot 10^{-5}$ [36]), and N denotes the total number of mutated positions. To incorporate the antiviral effect of LEN on a specific mutant strain i, we used equation (5).

### Probability of infection and mutant selection

During its elimination phase, LEN concentrations change very slowly. This means that LEN concentrations remain approximately constant at the timescale of infection establishment (or viral elimination), which is typically decided within a few days after virus challenge [42].

This time-scale separation allows to greatly simplify the computation of infection probabilities and mutant selection, akin to the methods used in [43] based on the strain-specific reproduction number.

The reproduction number $R_{0}(i)$ estimates the average number of infectious progeny produced by a (mutant) founder virus i during a single replication cycle. To simplify the representation, we define a drug-independent coefficient:

(12) $$\text{Λ}=\frac{\beta_{T}\cdot T_{u}}{\beta_{T}\cdot T_{u}+CL+CL_{T}\cdot T_{u}}\cdot \frac{k_{T}}{\delta_{PIC}+\delta_{T_{1}}+k_{T}},$$

which captures the efficiency of target cell infection and integration up to the virus production-competent compartment. In the absence of inhibitors, the reproduction number $R_{0}(i)$ for virus strain i is then given by:

(13) $$R_{0}(i)=\text{Λ}\cdot s(i)\cdot \frac{N_{T}}{\delta_{T2}},$$

where $\frac{N_{T}}{\delta_{T2}}$ represents the average number of infectious virus being produced from a late infected T-cell $\left(T_{2}\right)$. In line with classical results, $R_{0}<1$ implies that the infection dies out, whereas $R_{0}>1$ indicates that the virus replicates and may establish infection [44].

Based on the utilized viral dynamics parameters from [39], we computed $R_{0}(WT)\approx 11.8$ for the wild type WT and in the absence of drugs.

In the presence of LEN concentrations $C_{t}$, the instantaneous reproduction number of a viral strain i is then computed as

(14) $$R_{t}\left(C_{t},i\right)=R_{0}(i)\cdot \left(1-\eta \left(C_{t},i\right)\right).$$

which allows to integrate the PK/PD model (eqs. (1)-(3), eq. (5)). When LEN concentrations remain approximately constant over the duration of the infection event, the infection probability, after a single virus reaches a replication-competent environment, can be calculated directly from the instantaneous reproduction number, akin to [43]:

(15) $$P_{inf}\left(C_{t},i\right)=max\left(0,\text{Λ}\cdot \left(1-\frac{1}{R_{t}\left(C_{t},i\right)}\right)\right),$$

To calculate an average infection probability after homosexual virus exposure, the number of transmitted virions was drawn from a distribution relating donor virus loads to inoculum size in a replication-competent environment [45].

We calculated prophylactic efficacy as the relative HIV infection risk reduction relative to wild type virus challenge in the absence of LEN.

(16) $$\varphi_{i}\left({\text{S}}_{\text{LEN}}\right)=1-\frac{P_{inf}\left({\text{S}}_{\text{LEN}},i\right)}{P_{inf}(\emptyset ,WT)},$$

where $P_{inf}\left({\text{S}}_{\text{LEN}},i\right)$ denotes the probability of infection with mutant strain i in the presence of LEN and $P_{inf}(\emptyset ,WT)$ denotey the probabilitiy of infection with WT virus in absence of prophylaxis. This allows to assess the impact of fitness deficits s(i) of mutant viruses i, as well as the impact of LEN and any drug resistance phenotype simultaneously. I.e., in the absence of LEN, mutants may not spread as efficiently if they have fitness deficits, whereas at increasing LEN concentrations selective pressure may favor mutants over the wild type.

## Results

### Pharmacokinetics and pharmacodynamics of LEN

**Fig 2.**
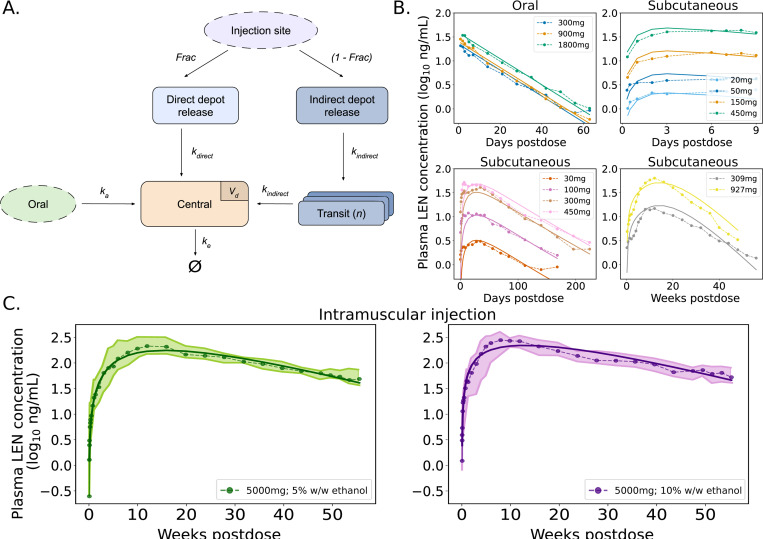
Pharmacokinetics of LEN A. Schematic representation of the developed PK-model for LEN. The model includes first-order absorption for oral dosing (tablets) and dual-release kinetics (direct and indirect depot release) for parenteral formulations, including subcutaneous (SC) and intramuscular (IM) injections. B. Model fitting results for LEN plasma concentrations after oral administration and three distinct SC formulations. Dots represent average concentrations observed in the respective clinical studies, and model predictions are shown as continuous lines based on best-fit parameters. C. Model-predicted PK profile for long-acting, next-generation intramuscular (IM) injection with LEN, formulated with 5% and 10% w/w ethanol. Dots show clinical median concentrations, and shaded areas represent the interquartile range (IQR; from the first to the third quartile). Clinical data used for the fitting are summarized in Table 1. Model performance was evaluated based on the reported PK properties listed in Table S1.

We fitted a single pharmacokinetic (PK) model (Fig. 2A) to concentration-time data from 15 dosing regimen, including 3 administration routes (oral, subcutaneous (SC) and intramuscular (IM)), Table 1. Predictions vs. average concentration-time profiles are shown in Fig. 2B-C indicating that the developed model appropriately captures average concentrations-time profiles across different doses and administration routes. The data and model highlight strong differences in drug uptake and distribution between oral and SC formulations, with a rapid uptake for oral regimen ($T_{\max}\approx 4-5$ hours) and a much longer time to peak concentrations for the different SC and IM formulations. Derived PK parameter values are summarized in Table 2, indicating slightly different parameter sets for the distinct formulations. Previously reported and model-predicted summary PK statistics ($C_{\max}$, $T_{\max}$ and $t_{1/2}$) agreed very well as shown in Table S1. Our modeling results highlighted that approximately 70.84% of the subcutaneously (SC) administered dose is released via an indirect absorption pathway (depot) in Studies 1 and 4. In Study 5 (IM formulation), the indirect release accounts for 86.78% for 5% w/w ethanol and 83.70% for 10% w/w ethanol. For Study 3, our model predicted only a fraction of 7.08% to be released via a depot, but this study is characterized by a very short observation time, indicating that a distinction between immediate, -vs depot release may not have been possible for this data set. Furthermore, LEN plasma concentrations may vary depending on the injection site and individual pharmacokinetic differences. Therefore, we extracted the possible range of injection-related variability from Phase III data and used it as the baseline variability in our analysis (see Supplementary Figure S1).

**Fig 3.**
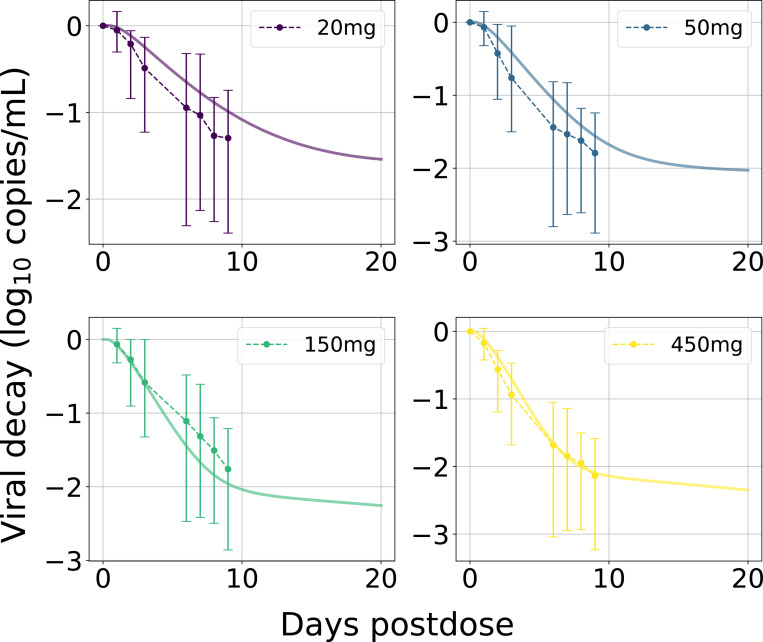
LEN viral decay in plasma with best-fit parameters. Average observed ‘change from baseline in HIV-1 RNA’ after single SC LEN injections (Study 3) are shown as dots with error bars representing minimum and maximum values. Model-predictions are shown as solid curves in the same color. The plasma VL (containing on average two viral RNAs [RNA/mL]) were calculated based on the total body virus, $V_{\text{total}}=\sum V_{I}+V_{NI}$, by assuming its distribution into plasma $\left(V_{\text{plas}}\right)$ and interstitial space $\left(V_{\mathrm{int}}\right)$, with the volume of distribution determined as $K_{\text{int:plas}}\times V_{\text{int}}+V_{\text{plas}}$, where $K_{\text{int:plas}}\approx 50$. For more details see [36].

Using the parameterized PK model, we then fitted LEN’s antiviral potency by estimating viral decay kinetics from a single dose monotherapy study in HIV infected individuals (Table 1; Study 3). Our parameter estimation resulted in an $IC_{50}$ of 1.9ng/mL for direct target inhibition and allowed to accurately approximate viral load kinetics across four SC dosing regimen for which data was available, Fig. 3. However, the data (n = 6 per dosing regimen) indicated large inter-individual variability in viral decay, as also implicated by a larger average viral decay for 50 vs. 150 mg dosing. All considered doses led to an at least 10-fold reduction in plasma HIV-1 RNA (log_10_ copies/mL) through day 10 [24].

### Analysis of mutant selection window

**Fig 4.**
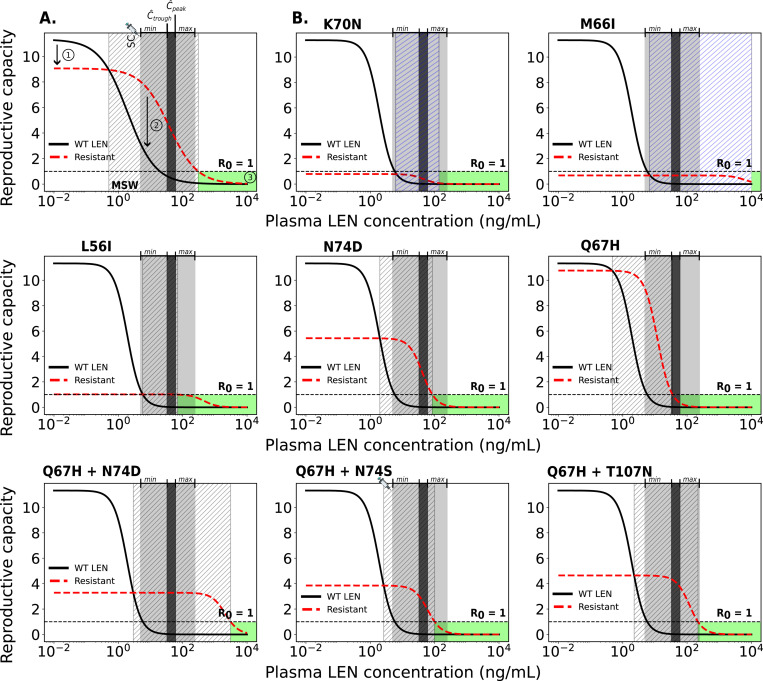
Relationship between plasma LEN concentrations after twice-yearly SC injections and viral reproductive capacity for wild type and resistant strains. A. Example showing the impact of a resistant strain on viral fitness (red dashed line: 20% lower replication capacity) and drug susceptibility (solid black line: 20-fold higher $IC_{50}$) compared to wild type (WT). Key features: (1) reduced fitness of the mutant, (2) the mutant selection window (MSW, diagonal hatching) where the mutant outcompetes WT, and (3) the green area where reproductive capacity is ≤ 1 (infection cannot occur). B. MSW analysis of five single and three double mutants. The horizontal dashed line at $R_{0}=1$ marks the threshold between viral suppression (below 1) and sustained replication (above 1). The dark gray areas indicate the clinically relevant population-average steady-state concentration ranges ($C_{trough}$-to-$C_{peak}$ concentration ranges) achieved by twice-yearly SC LEN injections. The light gray shaded areas represent ranges of inter-individual variability in drug concentrations informed by observations in the Purpose 2 trial (*min*-*max*). If the reproductive capacity of a mutant strain remains below 1 (blue shaded area, diagonal hatching), the viral strain is unable to reproduce and establish infection.

Next, we wanted to use the developed model to determine which concentrations of LEN may favor the selection of mutant strains. The selection of variants is influenced by fitness costs (i.e., selective disadvantage) and selection pressure through LEN which may favor the resistant virus at high concentration ranges. The mutant selection window (MSW) is characterized by $R_{t}\left(C_{t},WT\right)\leq R_{t}\left(C_{t},mut\right)>1$ [46], i.e. the mutant virus replicates better than wild type and it replicates to an extent that allows to sustain infection, compare Fig. 4A (hatched area). Both intrinsic mutant fitness (1−s(i)) and fold-change FC(i) in drug susceptibility determine the size of the MSW.

We used *in vitro* phenotypic parameters [28] to calculate the MSW of twice-yearly SC LEN for single and double mutants, as shown in Fig. 4B. According to the available *in vitro* parameters, single mutations such as K70N, M66I, and L56I have such low intrinsic fitness values that they cannot replicate at rates necessary to sustain infection, even in the absence of LEN (i.e. $R_{t}(\emptyset ,i)\leq 1$). In contrast, the single mutants Q67H and N74D show high to moderate fitness (1−s(i)=95% and 48%), respectively. Q67H exhibits moderate resistance but retains a replication capacity (low selective disadvantage) comparable to WT. According to our simulations, this mutation would be selected at LEN concentrations > 0.5ng/mL and would be sufficiently suppressed by concentrations exceeding 35.4ng/mL. The MSW of N74D ∈ [2, 85] ng/mL is confined by LEN concentrations achieved by twice-yearly SC injections (population-average $C_{\text{through}}$ to $C_{\text{peak}}=32-60\text{ng}/\text{mL}$; inter-individual variability: 4.8 − 240ng/mL). Regarding the single mutations, it is conceivable that Q67H may emerge under low drug pressure or pre-exist, with the additional N74D mutation appearing at higher concentrations or prolonged LEN exposure. The combination of both mutations leads to a highly resistant double mutant, as shown in Fig. 4B (lower panels). The corresponding MSW analysis for once-yearly intramuscular LEN injections is shown in the Supplementary Figure S2.

The evaluated double mutants all exhibited high levels of LEN resistance. Their MSWs (for Q67H+N74D, Q67H+N74S and Q67H+T107N) completely overlapped with the average steady-state concentrations observed following LEN injections.

Notably, any of the mutants (except K70N, M66I and L56I) is likely to become selected during the tail phase, if LEN injections were stopped.

### Prophylactic efficacy of LEN against transmission of WT and drug resistant virus

**Fig 5.**
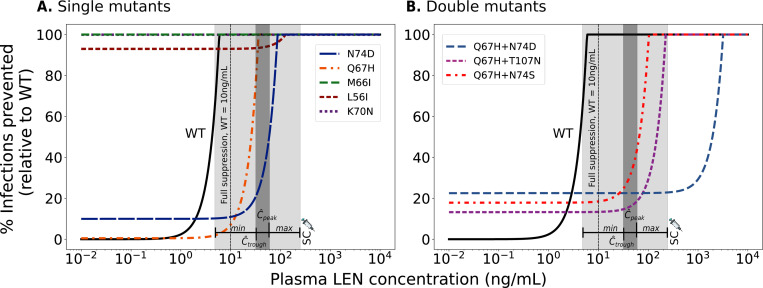
Reduction in HIV infection risk by twice-yearly SC LEN for wild type and mutant strains. Reduction in HIV wild type (WT) infection risk (black solid lines) and mutant viruses (colored non-solid lines) for A. single mutants and B. double mutants. Infection risk reduction (y-axis) with a particular variant and drug concentrations is computed relative to the infection risk with the WT in the absence of drug. The dark-gray area indicates the steady-state concentration range in an ‘average individual’ ($C_{trough}$-to-$C_{peak}$ concentrations) achieved with twice-yearly SC LEN injections. The light-gray area represent ranges of inter-individual variability in drug concentrations informed by observations in the Purpose 2 trial (*min*-*max*). Complete suppression of WT virus is achieved at a LEN concentration of 10ng/mL (dashed vertical line).

By coupling the direct effects of LEN (with inferred potency $IC_{50}=1.9\text{ng}/\text{mL}$) with the virus dynamics model (eqs. (1)-(3), eqs. (6)-(10)) we were able to estimate the relation between LEN concentrations and prophylactic efficacy, Fig. 5. We observed a steep concentration-prophylaxis relationship, similar to that observed for protease- and maturation inhibitors in earlier works [43]. This response curve does not have a sigmoidal shape like the Emax equation typically used to model concentration-effect relationships, and therefore complicates the estimation of 95% and 99% preventive concentrations directly from *in vitro* inhibitory concentrations, as done in [47].

When we calculated the drug concentration required to achieve 95% prophylactic efficacy $\left(EC_{95}\right)$ from the concentration-response curve depicted in Fig. 5, we obtained a value of $EC_{95}=5.8\text{ng}/\text{mL}$ against infection with wild type (WT) virus.

According to our model, at average steady-state LEN concentrations for twice-yearly SC, infection with WT virus would be fully averted.

Next, we investigated how LEN-based PrEP with twice-yearly SC administration may facilitate the transmission of drug-resistant viruses (corresponding analysis for once-yearly IM administration is provided in Supplementary Figure S3). We computed the reduction in HIV infection probability depending on the actual LEN concentration after virus exposure to a mutant strain i, relative to wild type in the absence of drug, eq. (16). This means that in the absence of drug, there may be a reduced HIV risk if a particular mutant strain has an intrinsic fitness disadvantage. At higher LEN concentrations, wild type infection may become less likely than infections with resistant strains, which increases the *relative proportion* of new infections with transmitted drug resistance.

Fig. 5 illustrates how both the fitness cost and resistance level of each strain affect the overall HIV risk reduction of LEN. Transmission of drug resistance is facilitated whenever the concentration-response curve of the the mutant lies below that of the WT (i.e. mutant transmission becomes more efficient). All investigated mutations (taken from [28]) show reduced susceptibility to LEN and may compromise the efficacy of PrEP. However, infection with the single mutant Q67H would be almost completely prevented at population-average steady-state drug levels achieved by SC injections, with a prophylactic efficacy > 70% (dark gray area), while the efficacy may drop to be as low as 3% in some individuals (lower variability limit; light gray area). Infection with the N74D mutant can still occur, but its overall infection probability is reduced by 20–40% in an ‘average individual’, compared with WT in the absence of LEN. Considering inter-individual PK variability (light gray areas in Fig. 5) reduction of infection risk may be as low as 10% in some individuals, which is solely attributed to intrinsic fitness disadvantage of the N74D mutation, rather than drug efficacy against it.

Infection with the double mutant Q67H+T107N cannot be efficiently prevented, showing only a 15–20% reduction in HIV risk across the full range of SC dosing concentrations. Infection with the Q67H+N74S double mutant is only poorly prevented with a 20–45% reduction in risk. In contrast, infection risks associated with the Q67H+N74D double mutant remain unaffected, both at average steady-state concentrations and across the full variability range observed with SC dosing.

In summary, these analysis highlight an elevated risk for transmitted drug resistance with the N74D single mutant at concentration rages typically observed during steady-state SC dosing and an elevated risk for all analyzed double mutants under twice-yearly SC-based PrEP.

### De novo emergence of drug resistant variants during LEN PrEP

While transmitted drug resistance increases the *fraction* of incident infections carrying drug resistance mutations, it may not necessarily increase the *absolute number* of individuals infected with resistant virus. I.e., if wild type infection would be fully prevented whereas infection with resistant virus would not be prevented at all, there would be the same *absolute number* of incident infections with resistant virus under LEN-PrEP. In contrast to transmitted resistance, de novo drug resistance emergence directly elevates the absolute number of individuals carrying drug resistant virus. Herein, we defined de novo drug resistance emerge as an event, where infection with a wild type virus occurs and where drug resistance is subsequently selected in the newly infected individual.

**Fig 6.**
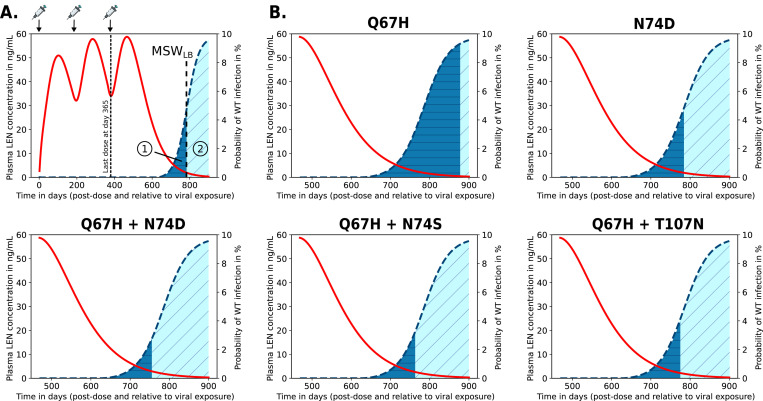
Quantification of de novo drug resistance emergence risk in scenarios where twice-yearly LEN SC doses are missed or when LEN-PrEP is stopped. Predicted average LEN plasma concentrations (red curve; left y-axis) after the first SC LEN injection and the probabilities of infection if exposure with WT virus occurred at the indicated time after the last LEN injection (blue dashed curve; right y-axis). A. Example of twice-yearly LEN SC dosing scenario, indicated at the top of the figure. If exposure with WT virus occurs after stopping LEN, two outcomes are possible: (i) infection with WT virus and de novo emergence of a resistant mutant (dark blue area), or (ii) infection with WT virus and selection of WT. The vertical line indicates the lower concentration threshold of the mutant selection window $\left(\text{MS}{\text{W}}_{LB}\right)$, i.e. at drug concentrations below this line WT will be selected (see Fig. 4). B. Simulation results for LEN-associated single and double mutants.

In particular, we looked at cases where individuals would stop taking LEN injections. In these scenarios, the long pharmacokinetic tail of LEN (Fig. 6A, red line) may give rise to concentrations that allow for infection with wild type virus and subsequently select drug resistance in the newly infected individual, Fig. 6A (dark blue areas). In our analyses in Fig. 6, we focus on twice-yearly SC injections, while Supplementary Figure S4 focuses on once-yearly IM injections.

For average drug concentrations observed during twice-yearly SC injections, we predicted that infection with WT virus can occur approximately 313 days (i.e. 45 weeks) after the last injection. If infection with WT virus occurs at that time all of the considered drug resistant single- and double mutants will subsequently be selected. Noteworthy, we also adapted the approach from [48] to verify the (relatively simplistic) predictions shown in Fig. 6, obtaining identical results. While de novo drug resistance emergence is certain for the indicated times in Fig. 6B (dark blue areas), there is still some residual HIV risk reduction against WT infection. Remarkably, the risk for de novo emergence of drug resistance after stopping twice-yearly SC injections is considerable at approx. 201, 105, 79, 86, 96 days after the last SC dose for mutations Q67H, N74D, Q67H+N74D, Q67H+N74S and Q67H+T107N. If infection with WT virus occurs after these time windows, resistance is unlikely to emerge (light blue areas). For IM once-yearly dosing, results are shown in Supplementary Fig. S4, indicating that de novo drug resistance emergence may actually happen approximately half a year to one year after the last LEN injection.

Next, we wanted to compare the relative likelihood of mutant de novo emerge during the LEN pharmacokinetic tail. We found that Q67H was the most likely de novo selected mutation, followed by N74D, Q67H+T107N, Q67H+N74S and Q67H+N74D. This order was identical for IM injections (Suppl. Fig. S4). This analysis also highlights that Q67H may emerge first, and then subsequently select +N74D, +N74S or +T107N.

Together these results hint at an important problem: De novo drug resistance emergence with LA-LEN PrEP can happen several months (up to one year for IM dosing) after the last injection, at a time where a former PrEP user may simply not be aware of these risks anymore. Noteworthy, while the moderately resistant single mutant Q67H is more likely to emerge de novo in individuals stopping PrEP, the highly resistant strains are more likely to be transmitted to individuals on PrEP (compare Fig. 5 and Fig. 6).

## Discussion

Lenacapavir (LEN), when injected subcutaneously every six months, has shown great promise as long-acting PrEP in the recently completed Purpose 1 (cis-gender women) and Purpose 2 (MSM and Transgender women) clinical phase trials [10, 14] and is endorsed by the WHO for HIV pre-exposure prophylaxis [49]. At the time of writing, once-yearly intramuscular injections are evaluated in clinical trials. Long-acting LEN formulations overcome the need for regular pill intake, which appears to be a major barrier to the success of oral PrEP in cis-gender women [8].

For example, the fraction of MSM not taking oral TDF/FTC-based PrEP was only 4–21% in major clinical studies like IPERGAY, HPTN 083, Purpose 2 and DISCOVER [11, 14, 50, 51], whereas it was 44, 64, 71 and ≈ 90% of cis-gender women in HPTN 084, Fem-PrEP, VOICE and Purpose 1 [10, 12, 52, 53]. Factors contributing to adherence differences between MSM and heterosexual women are not completely understood, but disbelieve in PrEP efficacy among cis-gender women [54], as well as stigmatization [55–57] may constitute barriers to oral PrEP uptake and adherence in women. LEN-based LA-PrEP may help overcome this adherence barrier in cis-gender women, who are most affected by HIV acquisitions globally [9]. However, long-acting agents remain unavailable or cost-prohibitive across much of the globe at the time of writing [58, 59], particularly as major funding programs are being stopped.

Another important and insufficiently researched aspect of LA-PrEP are potential risks associated with the emergence of resistant variants, which could limit their clinical scope and relevance for prevention. Notably, LEN has a low mutational barrier to drug resistance, with a single mutation conferring high-level, near-complete insusceptibility [24]. Unlike antiviral treatment which is taken life-long, PrEP denotes a voluntary choice of self-protection, whose (dis-)continuation may be influenced by the availability of insurance coverage, financial means, availability of the products, or perceived risk of HIV infection. Conditioned that PrEP is available, perceived risk denotes a strong predictor of PrEP uptake and continuation [60], but it poorly correlates with actual risk [61–65].

These observations hint that many individuals stopping PrEP for any of the above mentioned reasons may still be at risk of acquiring HIV. Since LEN-based LA-PrEP may persist up to 1–2 years after the last injection, drug concentrations may reside within the mutant selection window for a prolonged period. This constitutes a considerable risk for de novo drug resistance emergence if a person becomes infected after having stopped LEN-based LA-PrEP several months prior to virus exposure and infection.

In this work, we developed an integrated PK-PD, viral dynamics and evolution model to improve our understanding of LEN-based PrEP efficacy, and to evaluate the risks of drug resistance development in individuals with a history of LEN-PrEP use. Foremost, we derived a single PK model that allowed to predict oral, SC and IM dosing, for all publicly available, clinically tested LEN formulations. This model was coupled to an established viral dynamics model to predict LEN direct effects from Phase Ib clinical data. Using this integrated model, we were able to predict prophylactic efficacy for all investigated regimen. Further extension of the model with *in vitro* data allowed to predict the mutant selection window, risks for transmitted drug resistance, as well as the risks for de novo drug resistance emergence in individuals stopping LEN injections.

Considering both population-average steady-state drug concentrations (32–60ng/mL) and the inter-individual variability range (*min*-*max*: 4.8–240ng/mL), our model predicted that twice-yearly SC LEN PrEP remains fully protective against WT infection in the majority of individuals, with a small fraction of individuals not fully protected (Fig. S1). Furthermore, we observed limited protection against certain drug-resistant variants. Notably, two individuals in the LEN arm of the Purpose 2 study, both with sexually transmitted infections (STI, Syphillis, Clamydia), acquired HIV infection [14]. According to the analysis conducted in the Purpose 2 study, both infections may have occurred shortly after initiating LEN, possibly during the lead-in (i.e. pre-steady-state) phase. Interestingly, both individuals had viruses carrying drug resistance with the N74D mutation, which confers strong resistance *in vitro* [28] and is selected during LEN treatment [66]. While N74D alone implies a fitness deficit based on the *in vitro* data, we predicted it to be selected at high drug concentrations (> 2 ng/mL in Fig. 4). However, fitness deficits may in vivo be overcome by compensatory mutations [67], which may lead to their persistence, once selected.

In one infected individual in Purpose 2, LEN concentrations were consistently > 20ng/mL at week 4–13; -concentrations at which WT infection is unlikely (compare Fig. 5), arguing that infection may either have occurred at pre-steady-state in this individual or with transmitted drug resistance. The other individual infected in Purpose 2 had consistently lower than average concentrations of LEN (5–20ng/mL at weeks 4–26). According to our predictions, either (i) infection with WT occurred during the pre-steady-state phase, or (ii) infection with WT virus occurred at steady-state in this individual, followed by subsequent resistance selection, (iii) or resistance was transmitted. The dynamics of drug resistance emergence, in case of HIV infection occurs shortly before LEN application, or in the pre-steady-state phase may be similar to those observed during monotherapy, with distinct possible evolutionary trajectories involving any of the analyzed mutations alone or in combination [67]. Another possibility is that inter-individual variability with regards to within-host antiviral mechanisms (innate and adaptive immunity) may also contribute to variability in preventive plasma concentrations (e.g. $EC_{95}$, fully preventive concentrations), warranting further investigations.

Notably, resistance-associated mutations in the HIV capsid may be low in frequency in consensus sequences [68], but they are not absent in minority variants [69] and the HIV-1 capsid appears to be intrinsically variable [70]. Therefore, the occurrence of transmitted drug resistance may be low, but cannot be entirely ruled out (hypothesis iii). Infection with WT virus at steady-state may be unlikely (hypothesis ii), but also not impossible for the second individual.

A major limitation of our analysis is that due to the limited availability of individual data (see Table 1), we were not able to to fit and parameterize population pharmacokinetic models. To approximate inter-individual pharmacokinetic variability, we incorporated summary-level PK variability from the Purpose 2 trial (see Supplementary Figure S1), which highlighted that plasma concentrations may vary by approximately one order of magnitude between individuals. While this approach provides a reasonable estimate of concentration ranges, it cannot substitute for comprehensive Population-PK modelling. Nevertheless, our approach highlights that infections, albeit with small probability, may occur at steady-state twice-yearly LEN injections.

In our work, we predicted that the risk for de novo drug resistance emergence after wild type infection starts to rise when concentrations fall below 10ng/mL, which occurs approximately 8 months after the last injection of 927mg SC in an “average” individual, but may also occasionally be encountered during steady-state twice-yearly dosing, according to [14]. Based on utilized viral fitness parameters, we found that Q67H may be most frequently be selected in this scenario (due to its near-WT fitness). Interestingly, Q67H emerged in 2 participants receiving low SC doses (20mg and 50mg LEN) in a Phase Ib treatment study [71], which would be consistent with our predictions (Fig. 6) indicating that the Q67H mutation is preferentially selected at low LEN concentrations (> 0.5 ng/mL, Fig. 4). Noteworthy, while we predicted that the single mutant Q67H is more likely to emerge de novo in individuals stopping PrEP, it is not likely to be transmitted to individuals on PrEP (compare Fig. 5 and Fig. 6). I.e. transmitted drug resistance may favor strongly resistant variants, whereas de novo selection favors variants that confer little fitness cost.

Our simulations are based on mutant phenotype data (i.e. selective disadvantage and fold-change in $IC_{50}$) obtained from *in vitro* experiments. In vivo, mutant phenotypes may be influenced by the presence or pre-existence of compensatory mutations through epistatic interactions [72], partly explaining differences between *in vitro* and in vivo derived phenotypes [67], and potentially explaining distinct evolutionary trajectories of LEN failure through drug resistance development. For mathematical modeling, it is impossible to anticipate the different genetic backgrounds of susceptible viruses, which may subsequently favor one-over another evolutionary trajectory to drug resistance. However, utilized phenotypic parameters may under-predict the fitness of virus variants carrying the indicated mutations. In this context, the predictions made regarding the risk of de novo emergence of drug resistance may under-predict actual risks.

Together these results hint at an important problem: De novo drug resistance emergence with LA-LEN PrEP can happen several months (or years) after the last injection, at a time when a former PrEP user may not be aware of these risks anymore. These findings argue for public health considerations of the risks and benefits of LEN-based LA-PrEP and the need to develop LEN-PrEP discontinuation strategies.

Notably, the risk of de novo drug resistance emergence after PrEP discontinuation may similarly exist for long-acting cabotegravir, which also has a low barrier to drug resistance and which may persist month after discontinuation. In contrast, islatravir constitutes a very large genetic barrier to resistance and drug concentrations quickly decay after removal of ISL implants [13], which would minimize those risks.

Unlike long-acting PrEP, the risk of de novo drug resistance emergence during oral TDF/FTC-based PrEP may arise by different adherence mechanisms. The risk for resistance emergence after terminally stopping oral PrEP may be extremely low because of a high genetic barrier to drug resistance, as well as comparably short drug half-lives (i.e. ≈ 1.6 days for FTC-TP and ≈ 6.5 days for TFV-DP) [73]. These shorter drug half-lives may simply not provide enough “window of opportunity” for WT infection to subsequently select de novo drug resistance. Low constant levels of adherence, or frequent dis- and re-continuation may however create environments in which infection with WT can occur with subsequent drug resistance selection when oral TDF/FTC-based PrEP is mistakenly re-initiated after undetected infection (i.e. as monotherapy) [74].

## Supplementary Material

Supplement 1

## Figures and Tables

**Table 1.** Summary table of publicly available PK/PD data used for LEN. In total, 15 datasets were analyzed: 3 for oral administration, 10 for subcutaneous (SC), and 2 for intramuscular (IM) injections. All parenteral formulations showed distinct kinetics and were modeled separately.

Study ID [Ref.]	Study design/Focus	Formulation	Dataset
GS-US-200–4070 [24, 31] (Study 1)	Phase I in healthy subjects (*n* = 8/cohort). Safety, tolerability and SC PK.	SC aqueous suspension	30, 100 mg, 300 and 450 mg
GS-US-200–4071 [32] (Study 2)	Phase I in healthy subjects (*n* = 8/cohort). Effect of food on oral PK.	Oral tablets	300, 900 and 1,800 mg
GS-US-200–4072 [24] (Study 3)	Phase Ib in HIV-1 infected subjects (*n* = 6/cohort). Antiviral activity.	SC aqueous suspension	20 mg, 50 mg, 150 and 450 mg
GS-US-200–4358 [32, 33] (Study 4)	Phase I in healthy subjects (*n* = 8/cohort). Safety, tolerability and SC PK.	SC PEG/water solution	309 mg, 927 mg
Study 5 [27]	Phase I in healthy subjects (*n* = 20/cohort). Safety, tolerability and IM PK.	IM ethanol/ water solution	5000 mg with 5% w/w ethanol (F1), 10% w/w ethanol (F2)

**Table 2.** LEN mean PK parameter estimates.

Parameter	ID: Model estimates	Unit	Description
$k_{a}$	Study 2: 1.08	1/h	Oral absorption rate constant. Numerically obtained using Eq. (4).
$k_{direct}$	Study 1: 0.00037^†^ Study 3: 0.04 Study 4: 6 × 10^−5^ Study 5: 1.85 × 10^−4^	1/h	Direct absorption rate constant. Fixed [34] and optimized parameters.
$k_{indirect}$	Study 1: 0.0004 Study 3: 0.0017 Study 4: 0.0004 Study 5: 1.15 × 10^−4^	1/h	Indirect absorption rate constant. Parameters obtained by optimization.
Frac	Study 1: 20 Study 3: 2 Study 4: 20 Study 5: 1.52 (F1), 5.11 (F2)	%	Direct fraction of parenteral injections. Parameters obtained by optimization.
n	Study 1: 4^†^ Study 3: 1 Study 4: 2 Study 5: 1	-	Number of transit compartments. Fixed [34] and optimized parameters.
$V_{d}$ $V_{d}(300)$ $V_{d}(900)$ $V_{d}(1,800)$	139 13307 35394 46516	L	Volume of distribution for parenteral and oral dosing. Parameters obtained by optimization.
$k_{e}$	0.0026	1/h	Global elimination rate constant. Graphically determined from log-transformed oral PK data.

Fixed values are marked with † and were taken from [34]. We found that a power-law function f (dose) = 508.16 · dose^0.61^ with input dose in mg can be used to derive dose specific $V_{d}$ values. More details see section Methods.

## Data Availability

All analysis were performed using custom codes written in Python 3.12 and are available at https://github.com/KleistLab/LenPrEP under GPL 3.0 open access license. A frozen version of the code to reproduce all findings is available at Zenodo (https://doi.org/10.5281/zenodo.16612337).
