# Supplementary material for: Pharmacokinetics, pharmacodynamics, efficacy and drug resistance selection of injectable long-acting lenacapavir pre-exposure prophylaxis (PrEP) against HIV
Source: medRxiv. 2025 Sep 22:2025.08.26.25334527. Originally published 2025 Aug 28. Preprint. [Version 2] doi: 10.1101/2025.08.26.25334527 (PMC12407664; doi:10.1101/2025.08.26.25334527)
Supplement: Supplement 1 [file NIHPP2025.08.26.25334527v2-supplement-1.pdf]

medRxiv preprint doi: https://doi.org/10.1101/2025.08.26.25334527; this version posted September 22, 2025. The copyright holder for this preprint (which was not certified by peer review) is the author/funder, who has granted medRxiv a license to display the preprint in perpetuity.

It is made available under a CC-BY 4.0 International license .

Supporting Information

Clinical and model-based PK properties

**ID**	**Dose (in mg)**	**C_{max} (ng/mL)**		**T_{max} (days)**		**$t_{1/2}$ (days)**	
		study	model	study	model	study	model
SC aq. (Study 1)	30	3.2 ± 1.3	3.2	35.0 ± 7.3	28.3	35.5 ± 4.4	37.5
	100	14.7 ± 8.6	10.8	21.0 ± 12.5	28.5	30.3 ± 11.8	37.5
	300	47.9 ± 13.3	32.4	31.5 ± 8.8	25.5	43.1 ± 10	37.5
	450	58.4 ± 13.4	48.6	14.0 ± 11.7	29.4	39.9 ± 8	37.5
Oral (Study 2)	300	33.7 ± 32.5	21.2	0.2 ± 0.04	0.2	11 ± 2.6	11.1
	900	43.9 ± 32.2	28.5	0.2 ± 0.4	0.2	13.4 ± 2	11.1
	1,800	53.8 ± 25.8	34.2	0.3 ± 0.06	0.2	NA	11.1
SC aq. (Study 3)	20	2.5†	2.2	9.0†	3.3	0.7†	
	50	4.3†	5.4	7.0†	3.3	0.7†	
	150	15.0†	16.2	6.0†	3.1	0.7†	
	450	43.7†	48.6	8.0†	3.0	0.7†	
SC PEG (Study 4)	309	17.7 ± 8.9	17.2	98 ± 42.1	100.3	106 ± 43.2	62.8
	927	67.0 ± 36.7	51.6	77 ± 7.1	94.9	NA	62.8
IM (Study 5)	5000 (F1)	247.0 ± 81	176.2	84.1 ± 28.0	112.5	NA	96.3
	5000 (F2)	336.0 ± 120.4	219.8	69.9 ± 25.1	83.9	NA	96.3

Table S1. Overview of clinical PK properties and model-based calculations for oral, subcutaneous (SC) and intramuscular (IM) formulations. The reported PK values comprise C_{max} (maximal concentration), T_{max} (time to reach C_{max}) and $t_{1/2}$ (half-life). Values are taken from published clinical trial results or study protocols (GS-US-200-4334, GS-US-200-4072). Model-based calculations of the PK values are shown alongside the reported data. The values represent the mean (± std) of the considered doses, except for T_{max} and $t_{1/2}$, which are reported as the median (± Q3-Q1/2) with Q1 = first quartile and Q3 = thrid quartile. If clinical parameters were not available, PK values extracted from the data are reported and marked with a superscript †, otherwise, they are marked as NA (not available). Values that fall outside the reported range are shown in gray. All reported PK characteristics from clinical data and model-based calculations are in agreement (i.e., within reported variability), except for T_{max} of SC PEG (927 mg, Study 4) and C_{max} of the SC aqueous suspension (300 mg, Study 1).

medRxiv preprint doi: https://doi.org/10.1101/2025.08.26.25334527; this version posted September 22, 2025. The copyright holder for this preprint (which was not certified by peer review) is the author/funder, who has granted medRxiv a license to display the preprint in perpetuity.
It is made available under a CC-BY 4.0 International license .

Estimation of inter-individual variability in 927 mg SC LEN PrEP

To incorporate pharmacokinetic variability into our analysis, we digitized the published median, minimum, and maximum concentration profiles of the Purpose 2 study and expressed the lower and upper bounds as ratios relative to the median at each time point [14]. These concentration–time profiles represent a 10% subsample of participants who received 927 mg SC LEN, including two individuals in the LEN PK group who acquired HIV infection. Plasma concentrations were reported from week 4 to 52 (6 time points), covering two SC injections.

Since there were only minor differences between median and mean values (compare Supplementary Figure S1), the resulting scaling factors were applied to the 927 mg SC regimen (Study 4), which reported average concentrations following single-dose administration. Missing values (i.e., absorption and tail phases) in both concentration–time profiles were imputed by interpolation or extrapolation. To summarize the variability, we calculated the geometric mean of the factors, yielding 0.15 for the lower and 4.0 for the upper concentration limits, which we subsequently applied to the observed steady-state concentration range. Variability factors were also applied to other model simulations.

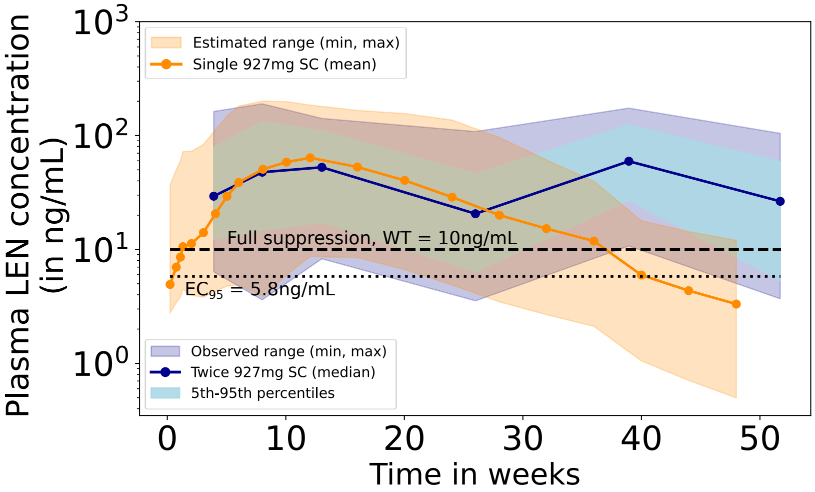

Figure S1. Inter-individual variability in LEN plasma concentrations after 927 mg SC administration. The blue line shows median concentrations in the pharmacokinetics cohort, with the light-blue shaded area indicating the 5th–95th percentiles and the dark-blue shaded area representing the minimum–maximum range observed in the Purpose 2 trial (twice-yearly SC dosing; dosing events at weeks 0 and 26). The orange line represents PK data after a single 927 mg SC dose (Study 4), including mean, minimum, and maximum concentrations, with imputed variability (derived by interpolation and extrapolation). A dashed horizontal line marks the concentration threshold for complete suppression of WT virus (10ng/mL LEN), and a dotted horizontal line indicates the concentration associated with 95% prophylactic efficacy ($EC_{95} = 5.8$ng/mL).

medRxiv preprint doi: https://doi.org/10.1101/2025.08.26.25334527; this version posted September 22, 2025. The copyright holder for this preprint (which was not certified by peer review) is the author/funder, who has granted medRxiv a license to display the preprint in perpetuity.

It is made available under a CC-BY 4.0 International license .

Mutant selection window (MSW) of once-yearly IM LEN PrEP

Using *in vitro* phenotypic parameters [28] we calculated the MSW of once-yearly IM LEN for single and double mutants, as shown in Supplementary Figure S2 (MSW for twice-yearly SC LEN see *Methods* and Fig. 4). Clinically relevant steady-state concentrations for IM LEN once-yearly were 62.57-238.59ng/mL (opulation-average), with a variability range of 9.4-954.4ng/mL. At average drug levels, Q67H would be fully suppressed, whereas the lowest concentration (9.4ng/mL) overlaps with its MSW ($[0.5, 35.4]$ ng/mL). The MSWs of N74D ($[2, 85]$ ng/mL) and Q67H+N74D ($[2.6, 100.15]$ ng/mL) overlap with average steady-state concentrations of once-yearly IM LEN. The MSWs of Q67H+N74D and Q67H+T107N completely overlapped with average steady-state concentrations.

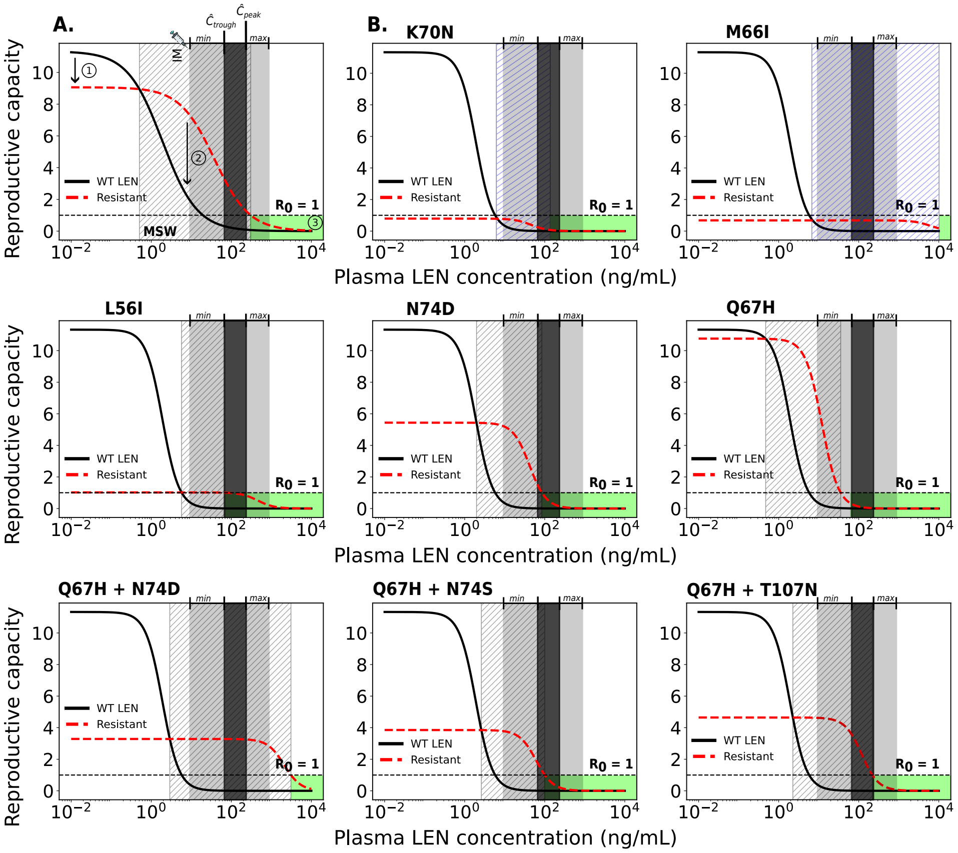

Figure S2. Relationship between plasma LEN concentrations after once-yearly IM injections and viral reproductive capacity for wild type and resistant strains. A. Example showing the impact of a resistant strain on viral fitness (red dashed line: 20% lower replication capacity) and drug susceptibility (solid black line: 20-fold higher IC_{50}) compared to wild type (WT). Key features: (1) reduced fitness of the mutant, (2) the mutant selection window (MSW, diagonal hatching) where the mutant outcompetes WT, and (3) the green area where reproductive capacity is ≤ 1 (infection cannot occur). B. MSW analysis of five single and three double mutants. The horizontal dashed line at $R_0 = 1$ marks the threshold between viral suppression (below 1) and sustained replication (above 1). The dark gray areas indicate the clinically relevant population-average steady-state concentration ranges (C_{trough}-to-C_{peak} concentration ranges) achieved by once-yearly IM LEN injections. The light gray shaded areas represent ranges of inter-individual variability in drug concentrations informed by observations in the Purpose 2 trial (*min-max*). If the reproductive capacity of a mutant strain remains below 1 (blue shaded area, diagonal hatching), the viral strain is unable to reproduce and establish infection.

medRxiv preprint doi: https://doi.org/10.1101/2025.08.26.25334527; this version posted September 22, 2025. The copyright holder for this preprint (which was not certified by peer review) is the author/funder, who has granted medRxiv a license to display the preprint in perpetuity.
It is made available under a CC-BY 4.0 International license .

Prophylactic efficacy of once-yearly IM LEN against WT and mutant HIV strains

As shown in the Supplementary Figure S3, infection with the WT virus would be completely avoided at the average steady-state LEN concentrations achieved by IM dosing once a year. We investigated how IM-based LEN PrEP (Phase III trial not yet initiated; NCT07047716) may facilitate the transmission of drug-resistant viruses (taken from [28]). Infection with the single mutant Q67H may occur at the lower variability limit (light gray area; ∼10% prevention) but would be completely prevented at average steady-state drug levels of IM dosing (dark gray area). Infection with the N74D mutant can also occur, but its overall infection probability is reduced by 50-100% on average, with a minimum reduction of at least 10% compared with WT in the absence of LEN. For double mutants, Q67H+T107N is not efficiently prevented at IM concentrations (15% infection-risk reduction at the lower variability range; ∼20% at C_{trough}) but would be fully prevented at peak steady-state drug levels (C_{peak}). The Q67H+N74S mutant is only partially suppressed, with ≥50% risk reduction at trough levels and complete prevention at peak IM levels. In contrast, infection risks associated with the Q67H+N74D double mutant are almost unaffected across the IM concentration range, with risk reductions of only 20-30%.

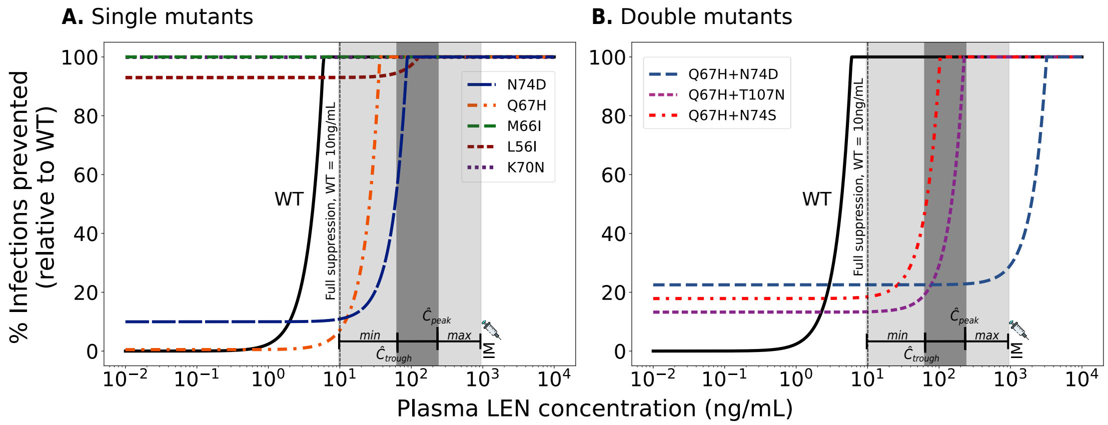

Figure S3. Reduction in HIV infection risk by IM LEN for wild type and mutant strains. Reduction in HIV wild type (WT) infection risk (black solid lines) and mutant viruses (colored non-solid lines) for A. single mutants and B. double mutants. Infection risk reduction (y-axis) with a particular variant and drug concentrations is computed relative to the infection risk with the WT in the absence of drug. The dark-gray area indicates the steady-state concentration range in an 'average individual' (C_{trough}-to-C_{peak} concentrations) achieved with once-yearly IM LEN injections. The light-gray area represent ranges of inter-individual variability in drug concentrations informed by observations in the Purpose 2 trial (*min-max*). Complete suppression of WT virus is achieved at a LEN concentration of 10ng/mL (dashed vertical line).

medRxiv preprint doi: https://doi.org/10.1101/2025.08.26.25334527; this version posted September 22, 2025. The copyright holder for this preprint (which was not certified by peer review) is the author/funder, who has granted medRxiv a license to display the preprint in perpetuity.

It is made available under a CC-BY 4.0 International license .

De novo emergence of resistant strains after LEN IM injection

To compare the relative likelihood of de novo mutation emergence, we considered a time window starting at day 965 and ending at day 1400 after the last LEN IM injection. Approximately 600 days after the last injection, infection with wild type virus becomes possible and may lead to the de novo emergence of Q67H, N74D, and all double mutants (see Supplementary Fig. S4). The estimated time points for de novo selection after the last IM dose were approximately 369, 198, 181, 164 and 151 days for Q67H, N74D, Q67H+T107N, Q67H+N74S and Q67H+N74D, respectively. Consequently, Q67H appears to be the most likely mutation to be selected, followed by N74D, Q67H+T107N, Q67H+N74S and Q67H+N74D. Identical results were obtained for twice-yearly SC LEN injections (see *Results*, Fig. 6).

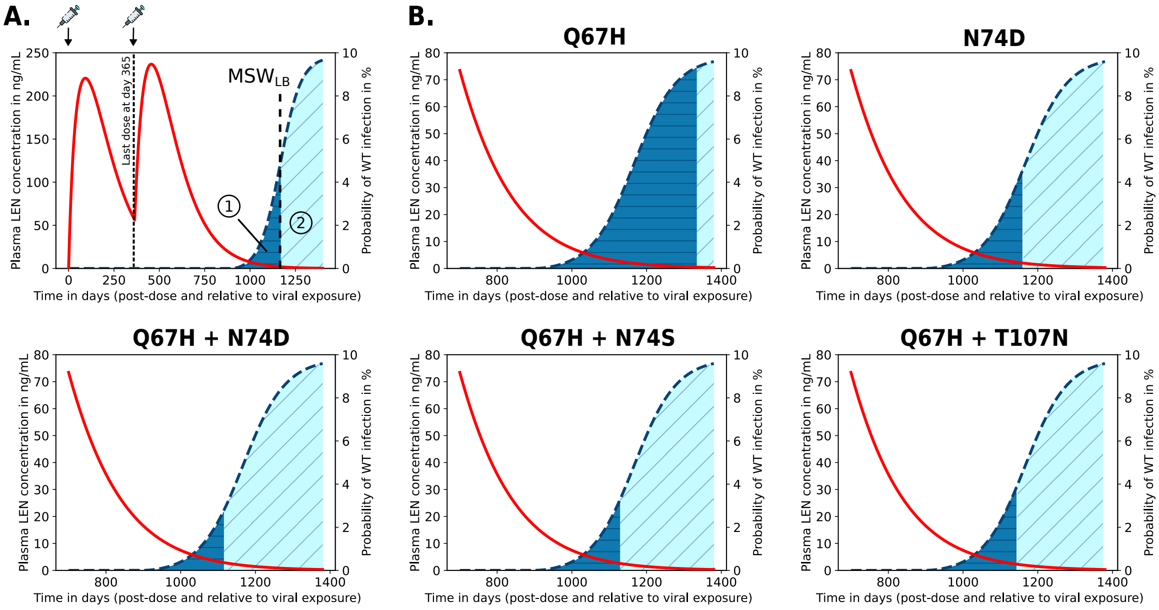

Figure S4. Quantification of de novo drug resistance emergence risk in scenarios where once-yearly LEN IM doses are missed or when LEN-PrEP is stopped. Predicted average LEN plasma concentrations (red curve; left y-axis) after the first IM LEN injection and the probabilities of infection if exposure with WT virus occurred at the indicated time after the last LEN injection (blue dashed line; right y-axis). A. Example of once-yearly LEN IM dosing scenario, indicated at the top of the figure. If exposure with WT virus occurs after stopping LEN, two outcomes are possible: (i) infection with WT virus and de novo emergence of a resistant mutant (dark blue area), or (ii) infection with WT virus and selection of WT. The vertical line indicates the lower concentration threshold of the mutant selection window (MSW_{LB}, compare Fig 4), i.e. at drug concentrations below this line WT will be selected. B. Simulation results for LEN-associated single and double mutants.
