# Supplementary material for: Pharmacokinetics, pharmacodynamics, efficacy and drug resistance selection of injectable long-acting lenacapavir pre-exposure prophylaxis (PrEP) against HIV
Source: medRxiv. 2025 Aug 28:2025.08.26.25334527. Preprint. [Version 1] doi: 10.1101/2025.08.26.25334527 (PMC12407664; doi:10.1101/2025.08.26.25334527)
Supplement: Supplementary file 1 [file NIHPP2025.08.26.25334527V1-supplement-1.pdf]

medRxiv preprint doi: https://doi.org/10.1101/2025.08.26.25334527; this version posted August 28, 2025. The copyright holder for this preprint (which was not certified by peer review) is the author/funder, who has granted medRxiv a license to display the preprint in perpetuity.
It is made available under a CC-BY 4.0 International license .

Supporting Information

Clinical and model-based PK properties

ID	Dose (in mg)	C_{max} (ng/mL)		T_{max} (days)		$t_{1/2}$ (days)	
		study	model	study	model	study	model
SC aq. (Study 1)	30	3.2 ± 1.3	3.2	35.0 ± 7.3	28.3	35.5 ± 4.4	37.5
	100	14.7 ± 8.6	10.8	21.0 ± 12.5	28.5	30.3 ± 11.8	37.5
	300	47.9 ± 13.3	32.4	31.5 ± 8.8	25.5	43.1 ± 10	37.5
	450	58.4 ± 13.4	48.6	14.0 ± 11.7	29.4	39.9 ± 8	37.5
Oral (Study 2)	300	33.7 ± 32.5	21.2	0.2 ± 0.04	0.2	11 ± 2.6	11.1
	900	43.9 ± 32.2	28.5	0.2 ± 0.4	0.2	13.4 ± 2	11.1
	1,800	53.8 ± 25.8	34.2	0.3 ± 0.06	0.2	NA	11.1
SC aq. (Study 3)	20	2.5†	2.2	9.0†	3.3	0.7†	
	50	4.3†	5.4	7.0†	3.3	0.7†	
	150	15.0†	16.2	6.0†	3.1	0.7†	
	450	43.7†	48.6	8.0†	3.0	0.7†	
SC PEG (Study 4)	309	17.7 ± 8.9	17.2	98 ± 42.1	100.3	106 ± 43.2	62.8
	927	67.0 ± 36.7	51.6	77 ± 7.1	94.9	NA	62.8
IM (Study 5)	5000 (F1)	247.0 ± 81	176.2	84.1 ± 28.0	112.5	NA	96.3
	5000 (F2)	336.0 ± 120.4	219.8	69.9 ± 25.1	83.9	NA	96.3

Table S1. Overview of clinical PK properties and model-based calculations for oral, subcutaneous (SC) and intramuscular (IM) formulations. The reported PK values comprise C_{max} (maximal concentration), T_{max} (time to reach C_{max}) and $t_{1/2}$ (half-life). Values are taken from published clinical trial results or study protocols (GS-US-200-4334, GS-US-200-4072). Model-based calculations of the PK values are shown alongside the reported data. The values represent the mean (± std) of the considered doses, except for T_{max} and $t_{1/2}$, which are reported as the median (± Q3-Q1/2) with Q1 = first quartile and Q3 = thrid quartile. If clinical parameters were not available, PK values extracted from the data are reported and marked with a superscript †, otherwise, they are marked as NA (not available). Values that fall outside the reported range are shown in gray. All reported PK characteristics from clinical data and model-based calculations are in agreement (i.e., within reported variability), except for T_{max} of SC PEG (927 mg, Study 4) and C_{max} of the SC aqueous suspension (300 mg, Study 1).

medRxiv preprint doi: https://doi.org/10.1101/2025.08.26.25334527; this version posted August 28, 2025. The copyright holder for this preprint (which was not certified by peer review) is the author/funder, who has granted medRxiv a license to display the preprint in perpetuity.
It is made available under a CC-BY 4.0 International license .

De novo emergence of resistant strains after LEN IM injection

To compare the relative likelihood of de novo mutation emergence, we considered a time window starting at day 965 and ending at day 1400 after the last LEN IM injection. Approximately 600 days after the last injection, infection with wild type virus becomes possible and may lead to the de novo emergence of Q67H, N74D, and all double mutants (see Supplementary Fig. S1). The estimated time points for de novo selection after the last IM dose were approximately 369, 198, 181, 164 and 151 days for Q67H, N74D, Q67H+T107N, Q67H+N74S and Q67H+N74D, respectively. Consequently, Q67H appears to be the most likely mutation to be selected, followed by N74D, Q67H+T107N, Q67H+N74S and Q67H+N74D. Identical results were obtained for twice-yearly SC LEN injections (see *Results*, Fig. 6).

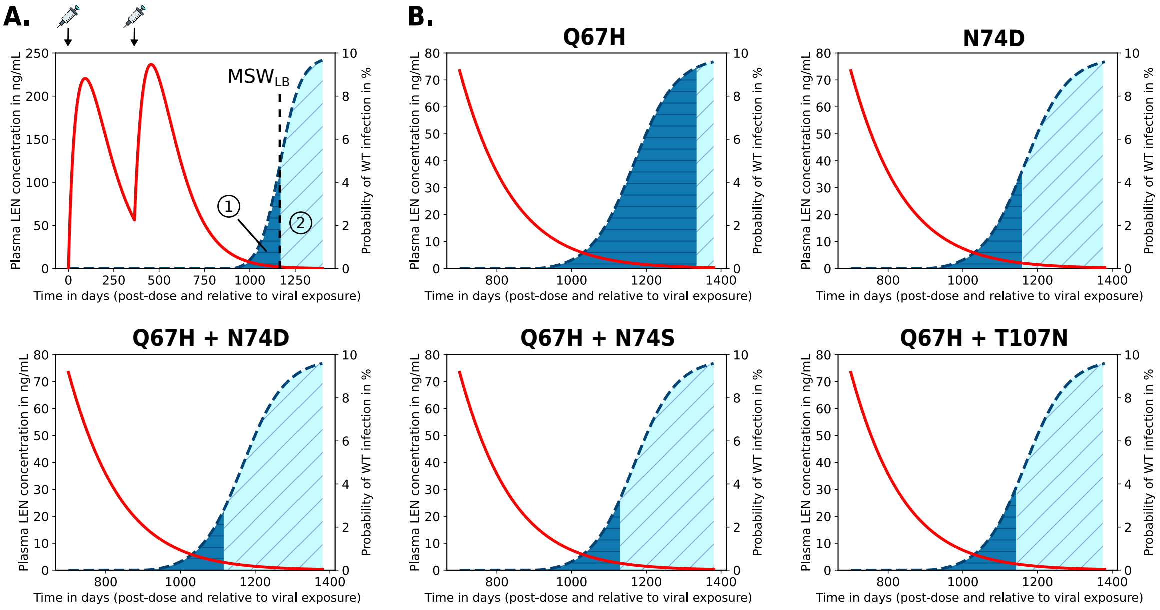

Figure S1. Quantification of de novo drug resistance emergence risk in scenarios where once-yearly LEN IM doses are missed or when LEN-PrEP is stopped. Predicted average LEN plasma concentrations (red curve; left y-axis) after the first IM LEN injection and the probabilities of infection if exposure with WT virus occurred at the indicated time after the last LEN injection (blue dashed line; right y-axis). A. Example of once-yearly LEN IM dosing scenario, indicated at the top of the figure. If exposure with WT virus occurs after stopping LEN, two outcomes are possible: (i) infection with WT virus and de novo emergence of a resistant mutant (dark blue area), or (ii) infection with WT virus and selection of WT. The vertical line indicates the lower concentration threshold of the mutant selection window (MSW_{LB}, compare Fig 4), i.e. at drug concentrations below this line WT will be selected. B. Simulation results for LEN-associated single and double mutants.
